# 

**Published:** 1872-05

**Authors:** 


### CONTENTS:

ORIGINAL COMMUNICATIONS:

ARTICLE I. Introductory to Lectures on Philosophy of Medicine, in the Spring Course of Rush Med. College, March 8, 1872 .......... 257

ART. II. Clinical Lectures on Diseases of the Ear .......... 275

ART. III. St. Luke's Hospital. Report of three cases .......... 278

ART. IV. Remarkable Surgical Case, with Operation by Prof. Freer, assisted by Prof. Powell, Cook County Hospital Clinic, May 10, 1872 .......... 281

ART. V. Removal of a Fibro-cellular Tumor from the Recto-Vaginal Septum .......... 283

ART. VI. Uræmic Poisoning .......... 287

SELECTIONS:

Intussusception, etc .......... 288

Clinical Lecture on the Significance of Uterine and Vaginal Discharges .......... 291

The proper Definition of the Word "Cure," as applied to Medicine .......... 296

Treatment of Spermatorrhœa .......... 299

AMERICAN MEDICAL ASSOCIATION. Proceedings of Twenty-third Annual Congress, held in Philadelphia, May 7-10, 1872 .......... 302

EDITORS' BOOK TABLE .......... 317

EDITORIAL .......... 320

OBITUARY .......... 320

